# Establishment and characterization of 18 Sarcoma Cell Lines: Unraveling the Molecular Mechanisms of Doxorubicin Resistance in Sarcoma Cell Lines

**DOI:** 10.1186/s12967-024-05700-y

**Published:** 2024-10-02

**Authors:** Young-Eun Cho, Soon-Chan Kim, Ha Jeong Kim, Ilkyu Han, Ja-Lok Ku

**Affiliations:** 1 Korean Cell Line Bank, Laboratory of Cell Biology, Cancer Research Institute, Seoul National University, Seoul, 03080 Korea; 2 Department of Biomedical Sciences, Seoul National University College of Medicine, Seoul, 03080 Korea; 3 Ischemic/Hypoxic Disease Institute, Seoul National University College of Medicine, Seoul, 03080 Korea; 4 Department of Orthopaedic Surgery, Seoul National University College of Medicine, 101, Daehak-ro, Jongno-gu, Seoul, 03080 Korea; 5 Laboratory of Cell Biology, Cancer Research Institute, Seoul National University College of Medicine, 101, Daehak-ro, Jongno-gu, Seoul, 03080 Korea

**Keywords:** Sarcoma, Doxorubicin, Drug resistance, Cell lines, Molecular profiling, Personalized medicine

## Abstract

Sarcomas, malignant tumors from mesenchymal tissues, exhibit poor prognosis despite advancements in treatment modalities such as surgery, radiotherapy, and chemotherapy, with doxorubicin being a cornerstone treatment. Resistance to doxorubicin remains a significant hurdle in therapy optimization. This study aims to dissect the molecular bases of doxorubicin resistance in sarcoma cell lines, which could guide the development of tailored therapeutic strategies. Eighteen sarcoma cell lines from 14 patients were established under ethical approvals and classified into seven subtypes. Molecular, genomic, and transcriptomic analyses included whole-exome sequencing, RNA sequencing, drug sensitivity assays, and pathway enrichment studies to elucidate the resistance mechanisms. Variability in doxorubicin sensitivity was linked to specific genetic alterations, including mutations in TP53 and variations in the copy number of genomic loci like 11q24.2. Transcriptomic profiling divided cell lines into clusters by karyotype complexity, influencing drug responses. Additionally, pathway analyses highlighted the role of signaling pathways like WNT/BETA-CATENIN and HEDGEHOG in doxorubicin-resistant lines. Comprehensive molecular profiling of sarcoma cell lines has revealed complex interplays of genetic and transcriptomic factors dictating doxorubicin resistance, underscoring the need for personalized medicine approaches in sarcoma treatment. Further investigations into these resistance mechanisms could facilitate the development of more effective, customized therapy regimens.

**Supplementary Information:**

The online version contains supplementary material available at 10.1186/s12967-024-05700-y.

## Introduction

Sarcomas, a diverse and challenging group of malignant tumors originating from mesenchymal tissues, remain difficult to treat effectively despite advances in therapeutic modalities such as surgery, radiotherapy, and chemotherapy [1–3]. Doxorubicin, a cornerstone chemotherapeutic agent in the management of sarcomas, is frequently hindered by the emergence of drug resistance, which significantly impacts treatment outcomes [4, 5]. Understanding the molecular mechanisms driving this resistance is essential for improving therapeutic strategies and patient prognosis [6].

Sarcoma cell lines serve as invaluable models for dissecting the complex interplay of genetic and epigenetic factors contributing to drug resistance [7, 8]. By leveraging these cell lines, researchers have made significant strides in elucidating the molecular mechanisms underlying resistance to doxorubicin [9, 10]. For instance, studies have identified alterations in drug influx and efflux transporters, DNA repair pathways, apoptotic signaling cascades, and drug metabolism enzymes as key determinants of doxorubicin resistance in various sarcoma subtypes [11, 12]. Nevertheless, the limited availability of sarcoma cell lines, coupled with the significant heterogeneity inherent in these tumors, underscores the need for the development and characterization of new cell lines that can better represent the diversity of sarcomas [7, 13–15].

This study addresses this gap by establishing 18 new sarcoma cell lines derived from 14 patients, with the aim of characterizing the molecular basis of doxorubicin resistance. These newly generated cell lines provide a valuable resource for investigating the mechanisms of drug resistance, particularly in the context of the varied genetic and transcriptomic landscapes observed across different sarcoma subtypes. The scientific value of these newly generated cell lines lies in their ability to capture the heterogeneity of sarcomas, especially at the molecular level. Through comprehensive molecular profiling, including whole-exome sequencing, RNA sequencing, and drug sensitivity assays, we aim to unravel the complex interplay of factors that contribute to doxorubicin resistance.

## Materials and methods

### Establishment and maintenance of human sarcoma cell lines

Eighteen cell lines were established from fourteen patients with written informed consent from all patients. The research protocol was approved by the institutional review board of the Seoul National University Hospital (IRB No. 1102-098-357). The study was performed in accordance with the Declaration of Helsinki.

A total of 14 tumor mass of bone and soft tissue sarcoma and adjacent 11 normal tissues were obtained from Seoul National University Hospital (Seoul, Korea). Each tumor was classified to 7 subtypes according to WHO diagnostic categories. Sarcoma samples were finely minced with scissors and dispersed into small aggregates by pipetting. Fine neoplastic tissue fragments were seeded into T-25cm^2^ flasks. Tumor cells were initially cultured in Opti-MEMI (Thermo Fisher Scientific, MA, USA) with 5% fetal bovine serum (FBS). After primary culture, SNU-5373, SNU-6035, SNU-6218 A, SNU-6219 A, SNU-6246 C, SNU-6349 A, SNU-6362, SNU-6766, SNU-6837, SNU-6938 and SNU-6962 cell lines were sustained in RPMI 1640 (Thermo Fisher Scientific, MA, USA) with 10% fetal bovine serum and 1% (v/v) penicillin and streptomycin (10,000U/ml). SNU-6036, SNU-6179B, SNU-6219B, SNU-6219E and SNU-6246D cell lines were sustained in Opti-MEMI with 5% fetal bovine serum and 1% (v/v) penicillin and streptomycin (10,000U/ml). The rest (SNU-6217 A and SNU-6380B) were sustained in Opti-MEMI with 5% fetal bovine serum and 1% (v/v) penicillin and streptomycin (10,000U/ml) and basic FGF (1ng/ml) and insulin (5ug/ml). Incubated flasks in humidified incubators at 37℃ in an atmosphere of 5% CO2 and 95% air.

### Growth properties and morphology in vitro

To obtain doubling time of each cell line, the density of 5 × 10^4^ to 2 × 10^5^ viable cells were seeded into 96 well white cell culture plate (SPL, #30196) with a volume of 100 µl, and cell viability was calculated daily for 10 days. Since the first cell seeding, in every 24 h, 10ul Cell-titer glo 2D (Promega, #G9241) solution was added to well of each seeded cell lines and the plate was incubated at room temperature for 10 min. Luminescence was measured with Luminoskan Ascent (Thermo Scientific) over 1000ms of integration time. Acquired growth rate values were calibrated with GraphPad Prism 5 (GraphPad Software, CA, USA). To observe the morphology of cell lines, phase-contrast microscopy was used. Mycoplasma contamination was identified by the 16 S-rRNA-gene-based polymerase chain reaction (PCR) amplification method using e-Myco Mycoplasma PCR Detection Kit (Intron Biotechnology, Gyeonggi, Korea).

### Genomic DNA/RNA extraction and DNA fingerprinting analysis

RNA and Genomic DNA extraction from resected tumor tissues and adjacent normal tissues and paired cell lines was performed using QIAamp DNA/RNA Mini kit (Qiagen). Genomic DNA extracted from each sarcoma cancer cell line was amplified using an AmpFlSTR identifiler Polymerase Chain Reaction (PCR) Amplification Kit (Applied Biosystems, CA, USA). A single cycle of PCR amplified 26 short tandem repeat markers (D3S1358, D1S1656, D2S441, D10S1248, D13S317, Penta E, D16S539, D18S51, D2S1338, CSF1PO, Penta D, TH01, Vwa, D21S11, D7820, D5S818, TPOX, D8S1179, D12S931, D19S433, D61043, D22S1045, DYS391, FGA, DYS576 and DYS570) and an Amelogenin gender-determining marker containing highly polymorphic microsatellite markers. Amplified PCR products were analyzed by an ABI 3500XL Genetic analyzer (Applied Biosystems).

### Fusion transcript detection by polymerase chain reaction (PCR)

To detect the fusion genes, complementary DNA was synthesized from total RNA. For cDNA synthesis, QuantiTect Reverse Transcription Kit (Qiagen) was used. One microgram of total RNA, 2 µL of gDNA Wipeout Buffer, and RNase free water up to 14 µL were mixed together and incubated at 42 °C for 2 min. The mixture was mixed with Quantiscript RT Buffer, RT Primer Mix, and Quantiscript Reverse Transcriptase and incubated at 42 °C for 45 min. Then, the mixture was incubated at 95 °C for 2 min and cooled down to room temperature. PCR was conducted using i-Taq™ DNA Polymerase (iNtRON, 25021) with 1 µL of cDNA. Amplification was performed at 94 °C for 30s, at optimal annealing temperature of each primer for 30s and 72 °C for 1 min for 35 cycles. Primers and annealing temperature of each primer are described below. Water was used as a negative control of each PCR. The PCR products were analyzed by electrophoresis on 2% agarose gels. The positive PCR products of CIC-DUX4 fusion transcript were gel purified from 2% agarose gel using the QIAquick Gel Extraction Kit (Qiagen Inc, Mississauga, ON).

**Table:** 

	Forward Primer	Reverse Primer	Annealing Temperature
SS18-SSX2	GGAGGATATAGACCAACACAG	CTGTTTTCTCTCACGCAG	62℃
CIC-DUX4	TTCAGGACCATGGCTTCTTCC	AAAGAAAGGCAGTTCTCCGC	62℃

### Sanger sequencing

PCR product was precipitated by 5% sodium acetate buffer (Sigma-Aldrich) and 95% ethanol mixed solution. Then washed product was set on ice for 10 min and centrifuged at 4℃, 14,000 rpm. Supernatant was discarded and the product was rinsed this time by 70% ethanol and centrifuged 14,000 rpm. Supernatant was discarded then the products were dried using vacuum concentrator (Eppendorf). 10 µL of distilled water was added to dilute precipitated sample. When the product is all diluted in distilled water, cyclic PCR was carried out. Two separate mixtures for forward and reverse sequences were made where they each include 5X sequencing buffer (Applied Biosystems), Big Dye (Applied Biosystems), forward or reverse primer, distilled water, and product from the previous step. Cyclic PCR was carried out with denaturation step at 96℃, annealing temperature at 55℃, and elongation at 60℃ for 25 cycles. The cyclic PCR product was then precipitated with 5% sodium acetate buffer and 95% ethanol mixed solution and set on ice for 10 min then it was centrifuged at 4℃ and supernatants were carefully discarded and the final product was dried using the vacuum concentrator. 10 µL Hi-Di formamide (Applied Biosystems) was added to dilute the dried product. This final product was transferred to 96 well PCR plate and denatured at 95℃ for 2 min before taken to 3500xL Genetic Analyzer (Applied Biosystems) for sequencing.

### Cell lines seeding/drug treatment procedure

2 × 10^5^ to 4 × 10^5^ viable cells from each cell line were seeded into well of 96 well white plate (SPL, #30196) in triplicate to measure sensitivity of several drugs. A day after, all cell lines were respectively treated for proper concentration of 6 drugs. After 72 h-incubation at 37 °C, 10ul Cell-titer glo solution was added to well of each seeded sarcoma cancer cell line. After 10 min-incubation at room temperature and an additional minute of shaking, luminescence was measured with a Luminoskan Ascent™ over 1000 ms of integration time. Data was normalized to vehicle and area under curve (AUC) values were calculated using R program version 3.6.3 (R Foundation for Statistical Computing, Vienna, Austria).

### Whole-exome sequencing

Whole-exome capture was performed on all 18 cell lines including paired tumor and normal tissues with the SureSelect Human All Exon V5 Kit (Agilent Technologies, Tokyo, Japan). The captured targets were subjected to sequencing using HiSeq 2500 (Illumina, San Diego, CA, USA) with the pair-end 100 bp read option for cell line samples and 200 bp read option for tissue samples. The sequence data were processed through an in-house pipeline. Briefly, paired-end sequences were first mapped to the human genome, where the reference sequence was UCSC assembly hg19 (original GRCh37 from NCBI, Feb. 2009) using the mapping program BWA (version 0.7.12), and generated a mapping result file in BAM format using BWA-MEM. Then, Picard-tools (ver.1.130) were applied in order to remove PCR duplicates. The local realignment process was performed to locally realign reads with BAM files reducing those reads identically matched to a position at the start into a single one, using MarkDuplicates.jar, which required reads to be sorted. By using the Genome Analysis Toolkit, base quality score recalibration (BQSR) and local realignment around indels were performed. Haplotype Caller of GATK (GATKv3.4.0) was used for variant genotyping of each sample based on the BAM file previously generated (SNP and short indel candidates are detected). Somatic mutations were identified by providing the reference and sequence alignment data of tumor tissues or cell lines to the MuTect2 (involved in GATK v3.8.0) with default parameters using tumor-normal mode. Those variants were annotated by SnpEff v4.1 g to vcf file format, filtering with dbSNP for the version of 142 and SNPs from the 1000 Genome Project. Then, SnpEff was applied to filter additional databases, including ESP6500, ClinVar, dbNSFP 2.9.

### Analysis of CNVs

For the detection of CNVs and copy-neutral loss of heterozygosity (CN-LOH) from exome sequencing data, we employed the CNV Radar [16]. The CNV score is defined as log2 fold change values of segment where IsCNV is True.

### Analysis of RNA sequencing

Paired end sequencing reads of cDNA libraries (101 bp) generated from a NovaSeq6000 instrument were verified with FastQC v 0.11.7. For data preprocessing, low quality bases and adapter sequences in reads were trimmed using Trimmomatic v 0.38. The trimmed reads were aligned to the human genome (UCSC hg19) using HISAT v2.1.0, a splice-aware aligner. Then, transcript assembly of known transcripts, novel transcripts, and alternative splicing transcripts was processed by StringTie v1.3.4d [17]. Based on the result, expression abundance of transcript and gene were calculated as read count or TPM value (Transcript per Million mapped reads) per sample. We applied FusionCatcher (version 1.00) for fusion gene analysis.

### Synergetic effect analysis

2 × 10^5^ to 4 × 10^5^ viable cells were plated in a 96-well white plate (SPL, #30196) in triplicate. A day after, cell lines were respectively treated with doxorubicin and Tiplaxtinin. Each drug was serially diluted at a ratio of 1:2 from 50µM to make 10 dose points. After incubation at 37 °C for 72 h, 10 µl Cell-titer glo solution was added to each well of each seeded cell line. After 10 min of incubation at room temperature and an additional minute of shaking, luminescence was measured with a Luminoskan Ascent™ over 1,000 ms of integration time. To test synergistic effects between doxorubicin and Tiplaxtinin, synergy scores were calculated by Bliss method using standard setting as implemented in SynergyFinder web application (version 3.0) [18].

### Western blot analysis

Cells were harvested with a cell scraper after washing with cold PBS three times. Cell pellets were washed with cold PBS three times. Whole protein was extracted with EzRIPA buffer (ATTO Co., Tokyo, Japan) supplied with 1% protease inhibitor (ATTO Co.) and 1% phosphatase inhibitor (ATTO Co.). Fractional protein harvest is performed using Subcellular Protein Fractionation Kit for Cultured Cells (Thermo Fisher Scientific) according to the manufacturers’ protocol. The volume of lysis buffer was adjusted to the number of cells collected in each vial. The protein concentration was determined by Pierce BCA Protein Assay Kit (Thermo Fisher Scientific). Mixture of equal amounts of protein, SDS buffer (Invitrogen, CA, USA), reducing buffer (Invitrogen), and distilled water was boiled at 98 °C for 10 min. Then, the mixture was loaded on 4–15% Mini-PROTEAN TGX Precast Gels (BIO-RAD, CA, USA) and blotted at 70 V for 2 h. Proteins were then transferred to Trans-Blot Turbo Transfer Pack (BIO-RAD) using Trans-Blot Turbo Transfer System V1.02 machine (BIO-RAD) at 2.5 Amp and 25 V. The membrane was incubated in 5% skim milk (BD biosciences, NJ, USA) containing 0.1% MgCl2, 10% TBS buffer, and 0.5% Tween 20 (VWR Life Science, PA, USA) for an hour at room temperature. The antibodies used in this study are as follows: anti-PAI-1 (Santa Cruz, sc-5297, 1:1000), anti-SMAD4(Santa Cruz, sc-7966, 1:1000), anti-ERK (cell signaling, #9102, 1:1000), anti-phospho ERK (cell signaling, #9101, 1:1000), anti-BCL2 (Abcam, ab59348, 1:1000), anti-cleaved PARP1 (Abcam, ab32561, 1:1000), anti-gamma H2A.X (cell signaling, #2577S, 1:1000), anti-beta actin (Santa Cruz, sc-130301, 1:1000). After washing with 5% skim milk three times, mouse or rabbit IgG secondary antibody (Jackson ImmunoResearch Labs, PA, USA) conjugated with peroxidase diluted in 5% skim milk solution (1:5000) was applied to membrane. SuperSignal West Pico PLUS Chemiluminescent Substrate (Thermo Fisher Scientific) was applied to the membrane. Protein levels were confirmed with ChemiDoc Touch Imaging System (Bio-Rad Laboratiores, CA, USA) using consecutive exposing mode with 1-min interval for 10 min.

### Statistical analysis

All statistical analyses were conducted and obtained using the R program version 4.1.0 (R Foundation for Statistical Computing, Vienna, Austria). Statistical analyses were conducted by Spearman’s correlation coefficient test and Wilcoxon rank-sum test (two-sided). A value of *p* < 0.05 was considered statistically significant. To determine the optimal number of clusters, we performed Elbow and Silhouette method using R package, NbClust (version 3.0). A Principal Component Analysis was conducted by FactoMineR (version 2.4) in R package.

## Results

### Sarcoma cell lines from various subtypes retained the histological patterns of original tumor

Eighteen sarcoma cell lines representing seven different subtypes were established from 14 patients who underwent surgery at Seoul National University Hospital. The subtypes included Undifferentiated Pleomorphic Sarcoma (UPS), Myxofibrosarcoma (MFS), Desmoplastic Small Round Cell Tumor (DSRCT), Synovial Sarcoma (SS), Chondrosarcoma (CHS), Pleomorphic Liposarcoma (PLS), Osteosarcoma (OS), and Fibrosarcoma (FS). Clinicopathological features of these sarcoma patients are summarized in Table 1. When the tumor mass was sufficiently large, the primary tumor was sectioned into multiple parts to account for the spatial heterogeneity of the sarcoma (SNU-6219 set and SNU-6246 set). Additionally, we longitudinally monitored a single sarcoma patient (SNU-5373; primary, SNU-6217 A; relapse) over a year to identify both molecular alterations and varying drug responses. DNA fingerprinting analysis of 26 tetranucleotide repeat loci and an Amelogenin gender-determining marker confirmed that each cell line was unique, without cross-contamination, and consistent with the primary tissue (Table S1).

**Table 1:** Clinicopathological information of 18 sarcoma cell lines

SNU Number	Sex/Age	Diagnosis	In Vivo				In Vitro	
			Size of tumor (cm)	Cellular morphology	Metastasis		Doubling Time (Day)	Cellular morphology
SNU-5373	M/81	MFS	2.7 × 1.5 × 0.8	spindle			7.989	polygonal
SNU-6035	M/85	UPS	5.5 × 5.0 × 2.3	spindle			3.431	fibroblast-like, polygonal
SNU-6036	F/58	UPS	2.4 × 2.0 × 1.2	spindle, pleomorphic			3.668	fibroblast-like
SNU-6179B	M/61	SS	16.0 × 6.5 × 3.5	spindle	Lung, Lymph Node		6.503	polygonal
SNU-6217 A	M/82	MFS	5.7 × 5.1 × 3.8	spindle, pleomorphic			6.382	fibroblast-like, polygonal
SNU-6218 A	F/67	CHS	10.5 × 3.5 × 3.3	chondrocytic			3.015	polygonal
SNU-6219 A	F/75	UPS	22.0 × 14.5 × 9.0	spindle, pleomorphic	Lung, Lymph Node		5.794	fibroblast-like
SNU-6219B	F/75	UPS	22.0 × 14.5 × 9.0	spindle, pleomorphic	Lung, Lymph Node		4.002	polygonal
SNU-6219E	F/75	UPS	22.0 × 14.5 × 9.0	spindle, pleomorphic	Lung, Lymph Node		7.175	fibroblast-like
SNU-6246 C	M/45	DSRCT	6.2 × 5.0 × 5.0	round			5.286	polygonal
SNU-6246D	M/45	DSRCT	6.2 × 5.0 × 5.0	round			5.041	polygonal
SNU-6349 A	M/59	MFS	10.0 × 6.4 × 5.3	spindle, pleomorphic	Lung		5.101	fibroblast-like, polygonal
SNU-6362	F/43	PLS	7.2 × 2.8 × 1.5	spindle, pleomorphic, lipoblastic	Lung, Brain, Small Bowel		3.917	fibroblast-like, polygonal
SNU-6380B	F/72	DSRCT	5.2 × 4.5 × 2.5	round, epithelioid			5.735	fibroblast-like, polygonal
SNU-6766	M/74	UPS	15.5 × 15.1 × 4.8	spindle, pleomorphic, giant	Lung		4.612	fibroblast-like, polygonal
SNU-6837	M/61	OS	8.3 × 5.5 × 3.5	spindle	Lung, Bone		4.439	fibroblast-like
SNU-6938	M/41	FS	8.6 × 6.7 × 5.5	spindle			6.022	fibroblast-like, polygonal
SNU-6962	F/53	MFS	Not Available	Not Available			4.265	fibroblast-like

MFS = Myxofibrosarcoma, UPS = Undifferentiated Pleomorphic Sarcoma, SS = Synovial Sarcoma, CHS = Chondrosarcoma, DSRCT = Desmoplastic Small Round Cell Tumor, PLS = Pleomorphic Liposarcoma, OS = Osteosarcoma, FS = Fibrosarcoma

**Fig. 1:**
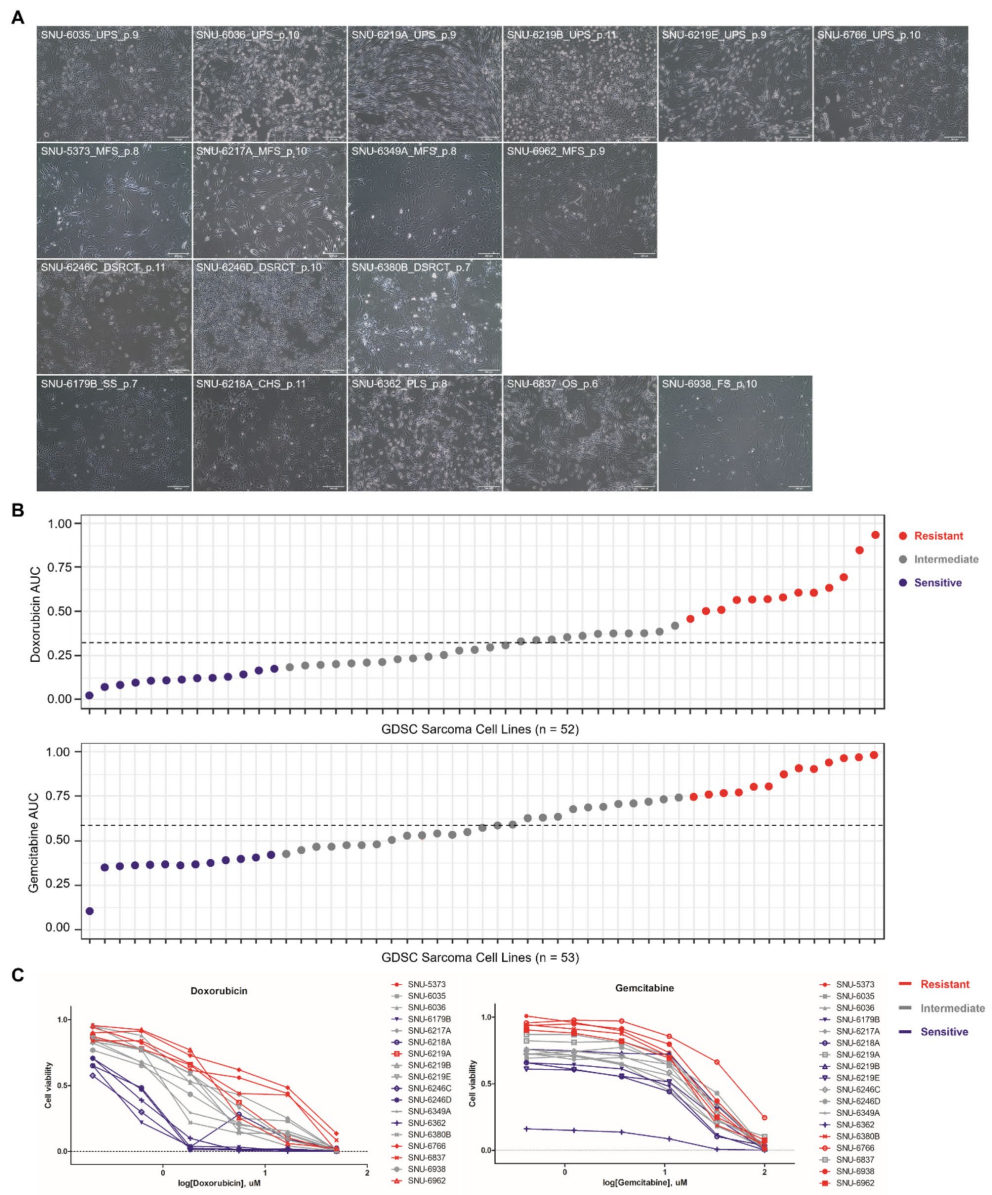
**Morphological Characteristics and Drug Response Patterns of Established Sarcoma Cell Lines. See also Figure ****S1****A-B and Table S2B-C. (A)** Representative images of the 18 sarcoma cell lines. Scale bar = 250 µM **(B)** Genomics of Drug Sensitivity in Cancer (GDSC) data illustrates the distribution of Area Under the Curve (AUC) values for Doxorubicin (*n* = 52) and Gemcitabine (*n* = 53) across various sarcoma subtypes. Dotted line represents the median. The lower quartile (Q1) and the upper quartile (Q3) were classified as sensitive and resistant respectively. The others were considered as intermediate. **(C)** The response of 18 SNU sarcoma cell lines to Doxorubicin and Gemcitabine is presented through AUC values. A clearer segregation between resistant and sensitive cell lines is noted in response to Doxorubicin, corroborating the GDSC data. This indicates a distinct classification potential of sarcoma cell lines based on their response to Doxorubicin. (Red; Resistant, Grey; Intermediate, Blue; Sensitive)

The morphologies of the established 18 cell lines are primarily categorized into fibroblast-like and polygonal shapes, with the majority being fibroblast-like (Fig. 1A). Spatial heterogeneity was noted in the SNU-6219 set, where the tumor mass from a single patient was divided into three sections and cultivated independently. SNU-6219 A and SNU-6219E exhibited typical fibroblast-like shapes, while SNU-6219B displayed a polygonal shape, suggesting that the original sarcoma mass contained various cellular populations, consistent with previous findings. Most cell lines retained their pathological morphologies (Table 1), though some underwent changes during consecutive passaging, altering their morphologies in vitro. For example, SNU-6246 C and SNU-6246D were identified as round cell tumors, yet they exhibited polygonal shapes as the subculture progressed (Fig. 1A).

### Sarcoma cell lines exhibited separated pattern in responses to Doxorubicin

Sarcoma presents a formidable challenge to oncological treatment due to its heterogeneity. Among the pharmacotherapies utilized for sarcoma, Doxorubicin and Gemcitabine have emerged as significant chemotherapeutic agents, albeit with distinct mechanisms of action [19, 20]. We first screened 18 established SNU sarcoma cell lines encompassing various subtypes utilizing six clinically used drugs: Dacarbazine, Docetaxel, Doxorubicin, Gemcitabine, Ifosfamide, and Pazopanib [21, 22]. Two cell lines, SNU-6218 A (CHS) and SNU-6362 (PLS), demonstrated a notably good response to Docetaxel, Doxorubicin, and Gemcitabine. The other cell lines mostly exhibited resistance to all drugs except for Doxorubicin, with sensitivity clustering around Doxorubicin (Figure S1A, Table S2A). Using the Genomics of Drug Sensitivity in Cancer (GDSC) Sarcoma cell line data [23], we evaluated the overall response rates across different sarcoma types. Certain rare subtypes, such as Alveolar rhabdomyosarcoma and Embryonal rhabdomyosarcoma, were omitted from our analysis due to the singular cell line representation for these categories. Across the board, Ewing sarcoma, Leiomyosarcoma, Myxofibrosarcoma, and Synovial sarcoma demonstrated comparable response rates to both Doxorubicin and Gemcitabine. However, the Desmoplastic small round cell tumor and Fibrosarcoma subtypes exhibited a more favorable response to Doxorubicin (Figure S1B). Within the GDSC sarcoma cell lines, a discernible trend of Area Under the Curve (AUC) distribution was observed for both Doxorubicin and Gemcitabine (Fig. 1B, Table S2B and S2C). Notably, the median AUC for Doxorubicin was significantly lower compared to Gemcitabine. This data pattern indicates a generally enhanced response to Doxorubicin among the sarcoma cell lines within the GDSC dataset. Corroborating this finding, our research involving 18 sarcoma cell lines also revealed median AUC for Doxorubicin was lower as shown in Fig. 1C. Additionally, a more distinct segregation between resistant and sensitive strains in response to Doxorubicin versus Gemcitabine was observed (Fig. 1C). It is postulated that the molecular attributes of the sarcoma cell lines could be more distinctly classified based on their response to Doxorubicin as opposed to Gemcitabine.

### Categorization and genetic analysis of sarcoma cell lines based on Doxorubicin sensitivity

**Fig. 2:**
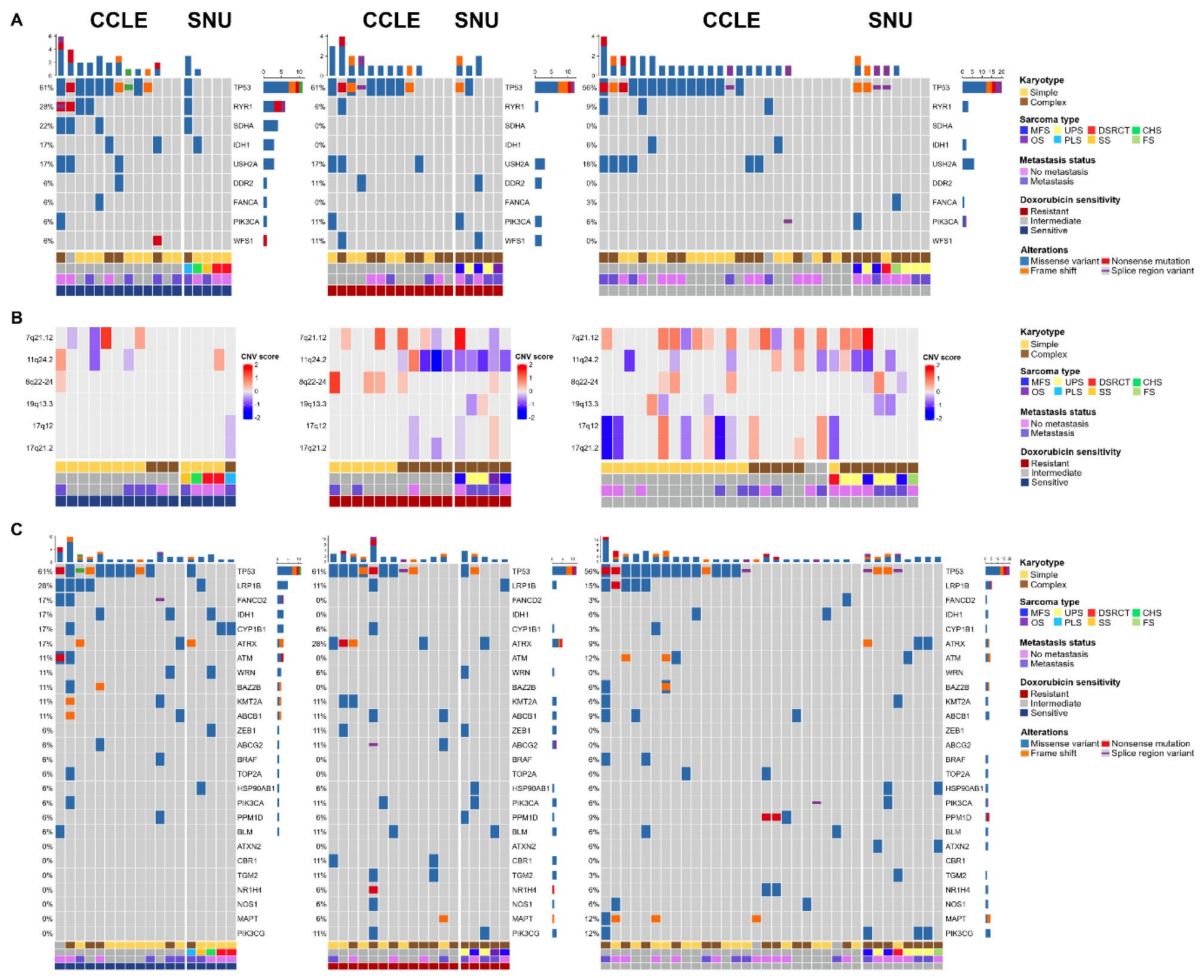
**Categorization and Genetic Analysis of Sarcoma Cell Lines Based on Doxorubicin Sensitivity. See also Figure S2A-B**,** Figure S3A-D**,** and Table S3A-E. (A)** Classification of sarcoma cell lines into Doxorubicin sensitivity categories (sensitive: 0–25%, intermediate: 26–75%, resistant: 76–100%) reveals a correlation between karyotype complexity and Doxorubicin resistance. Doxorubicin-resistant cell lines predominantly exhibit TP53 mutations, while sensitive cell lines display a broader range of mutations, including RYR1, IDH1, and SDHA. CCLE cohort and SNU cohort were distinguished with wider space. **(B)** Copy number variation (CNV) analysis indicates that Doxorubicin-resistant sarcoma cell lines have more aberrant CNVs compared to sensitive ones across both CCLE and SNU cohorts, suggesting a link between CNV at this locus and drug resistance. The genomic locus 11q24.2 shows a low CNV score in resistant cell lines, which correlates with Doxorubicin resistance. **(C)** Mutational analysis of 26 genes related to Doxorubicin sensitivity demonstrates that sensitive cell lines tend to harbor more mutations. The deletion of 11q24.2 and its impact on gene expression and Doxorubicin response is also highlighted, showing statistical correlations but no significant impact on patient survival in the TCGA sarcoma cohort. Classifying factors including karyotype, sarcoma type, metastasis status, and doxorubicin sensitivity are indicated below. Different types of alterations are presented with representative colors

We subsequently categorized sarcoma cell lines based on their response rate to Doxorubicin sensitivity. According to area under the curve (AUC) values, those within the 0–25% range were classified as sensitive, 26–75% as intermediate, and 76–100% as resistant. Additionally, we differentiated sarcoma cell lines within the Doxorubicin groups by clinicopathological features such as karyotype, sarcoma type, and metastasis status. We then accessed mutational profiles of Sarcoma cell lines using whole-exome sequencing (WES). It has been consistently reported that tumor cell lines can recapitulate the genetic alterations of the original tumors [24, 25]. Given the limited tumor mass obtained from surgical resection, we selected eight tumor tissue-cell line pairs to validate that our cell lines retained most of the mutations and copy number variations (CNVs) of the original tumors. Overall, nearly 90% of the mutations were shared between tumor tissues and cell lines (Figure S2A). We also compared variant allele frequencies (VAFs) of each mutation to confirm that the clonal composition was maintained in cell lines during consecutive cultures. All eight pairs displayed a correlation coefficient (R) > 0.84, suggesting that the mutational population of the original tumor tissue was largely retained in the cell lines (Figure S2A). The overall scatter patterns of VAFs indicated that mutations were being enriched in cell lines, implying that continuous passaging might apply selective pressure favoring certain mutational clones (Figure S2A). We also confirmed that the pattern of CNVs was maintained in the cell lines (Figure S2B). No specific variation was found in spatially heterogeneous samples. Among the frequently mutated genes in Sarcoma, *TP53* was the most commonly mutated, exhibiting a range of mutation types including missense variants, nonsense mutations, frameshifts, and splice region variants (Fig. 2A). Mutation frequencies and types varied according to Doxorubicin sensitivity, displaying distinct patterns for sensitive (blue), resistant (red), and intermediate (grey) responses. Doxorubicin-resistant cell lines predominantly featured mutations in *TP53*, whereas Doxorubicin-sensitive cell lines exhibited a broader array of mutations in genes such as *RYR1*, *IDH1*, and *SDHA*, which were frequently observed in Doxorubicin-sensitive cell lines across both Cancer Cell Line Encyclopedia (CCLE) and SNU cohorts (Fig. 2A). Other clinicopathological factors, such as metastasis status or sarcoma subtypes, were infrequently associated with the mutational patterns of *TP53*, *RYR1*, *IDH1*, and *SDHA*. There was a correlation between Doxorubicin sensitivity and the karyotype of sarcoma cell lines, with complex sarcoma types showing a more Doxorubicin-resistant pattern (Fig. 2A, Table S3A and S3B). We also analyzed the copy number variation (CNV) of selected genomic loci associated with Doxorubicin resistance. Overall, Doxorubicin-resistant sarcoma cell lines exhibited more aberrant CNVs compared to their Doxorubicin-sensitive counterparts (Fig. 2B). Specifically, the genomic locus 11q24.2 frequently demonstrated a low CNV score in both CCLE and SNU cohorts (Fig. 2B, Figure S3A and Table S3C). We further analyzed the gene-drug interactions using the Wilcoxon rank-sum test, which identified eight statistically significant interactions. Among these, the deletion of 11q24.2 was strongly correlated with resistance to Doxorubicin (Figure S3B, S3C and Table S3D). Additionally, the Pearson correlation between the CNV score of 11q24.2 and the AUC of Doxorubicin demonstrated a significant inverse relationship (Figure S3C). We selected nine representative genes located within the 11q24.2 locus and calculated the correlations between their mRNA expression, Doxorubicin response, and the CNV of 11q24.2 (Table S3E). The results indicated that the mRNA expressions of PUS3, EI24, FAM118B, RPUSD4, and FOXRED1 were statistically correlated with both Doxorubicin response and the CNV of 11q24.2. Despite these statistical significances, there was no observed difference in patient survival based on the mRNA expression levels of these genes, according to the TCGA sarcoma cohort (Figure S3D). Additionally, we assessed the mutational status of 26 genes known to influence Doxorubicin sensitivity. Mutation frequency was seldom categorized into specific mutational patterns, with Doxorubicin-sensitive cell lines tending to harbor more mutations (Fig. 2C). In summary, genomic variation seldom influenced Doxorubicin resistance in both the CCLE and SNU cohorts.

### Transcriptomic analysis and pathway enrichment in SNU sarcoma cell lines reveal karyotype-linked Doxorubicin sensitivity

**Fig. 3:**
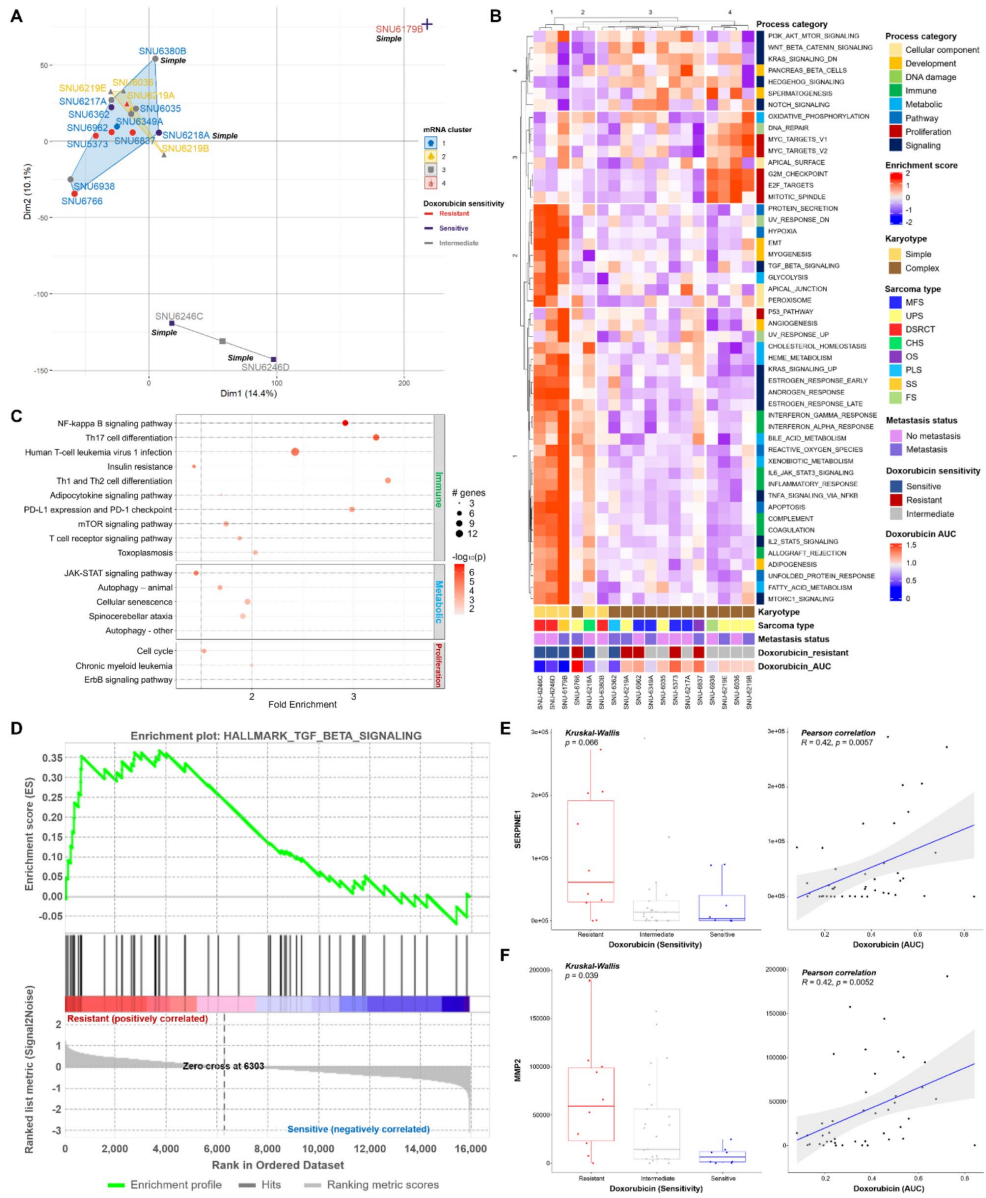
**Transcriptomic Clustering and Pathway Enrichment in SNU Sarcoma Cell Lines Linked to Doxorubicin Sensitivity. See also Figure S4A-E and Table S4A-E. (A)** Principal component analysis (PCA) of mRNA expression profiles categorizes SNU sarcoma cell lines into four distinct clusters. Cell lines classified with simple karyotypes are marked with italic “Simple”, while the rest are complex karyotypes. Each mRNA cluster is marked with representative colors. The X-axis indicates dimension 1 (14.4% of total mRNA), and the Y-axis indicates dimension 2 (10.1% of total mRNA). **(B)** Single sample gene set enrichment analysis (ssGSEA) using the HALLMARK gene set database identifies distinct pathway enrichment patterns. Classifying factors including karyotype, sarcoma type, metastasis status, and doxorubicin sensitivity are indicated below. The process category of each HALLMARK pathway is indicated on the right side of the heatmap. **(C)** Protein-protein interaction network (PIN) analysis highlights significant enrichment of various pathways. The size of the dot correlates with the number of genes counted within the pathway. **(D)** The TGF-β signaling pathway is enriched in sarcoma cell lines, with expressions of SERPINE1 and MMP2 shown. **(E)** Expression analysis of SERPINE1 and **(F)** MMP2 genes in sarcoma cell lines, with p-values indicated above the graph. Kruskal-Wallis test and Pearson correlation coefficient are used to estimate p-values

We classified the transcriptomic patterns of SNU sarcoma cell lines. The batch effect between GDSC and SNU sarcoma cell lines was too pronounced to be removed. Principal component analysis (PCA), encompassing the entire mRNA expression profile, revealed four clusters. Clusters 1 and 2 predominantly consisted of cell lines with complex karyotypes. Clusters 3 and 4, composed of a smaller number of cell lines, showcased transcriptomic differences indicative of simple versus complex karyotype sarcomas. Four out of five doxorubicin-resistant sarcoma cell lines were grouped within cluster 1, yet there was no significant dimensional distance from cluster 2, suggesting that more specific pathways may account for the diverse responses to doxorubicin (Fig. 3A and Table S4A). We also analyzed fusion genes using RNA sequencing data. The SS18-SSX2 fusion gene was identified in SNU-6179B, while the CIC-DUX4 fusion gene was found in SNU-6246 C and SNU-6246D (Figures S4A, 4B and Table S4B). As each fusion gene was identified in only a single sample, their presence was not correlated with responses to Doxorubicin. We subsequently performed single sample gene set enrichment analysis (ssGSEA) using the HALLMARK gene set database [26]. The enrichment scores were subjected to k-means clustering to determine if the responses to doxorubicin could segregate specific pathway categories (Fig. 3B and Table S4C). Once again, the majority of sarcoma cell lines exhibiting favorable responses to doxorubicin displayed simple karyotypes. The type of sarcoma and metastasis status rarely influenced the response to doxorubicin. SNU-6766, classified with a complex karyotype, demonstrated the most resistant response to doxorubicin. Overall, signaling processes involving WNT_BETA-CATENIN and HEDGEHOG signaling were downregulated in doxorubicin-sensitive sarcoma cell lines, and upregulated in doxorubicin-resistant sarcoma cell lines. Additionally, immune and metabolic categories were generally enriched in doxorubicin-sensitive sarcoma cell lines. We integrated a protein-protein interaction network (PIN) to identify distinct subnetworks used for clustering in enrichment analyses. In concurrence with previous ssGSEA results, immune, metabolic, and proliferation pathways were significantly enriched in doxorubicin-resistant sarcoma cell lines compared to doxorubicin-sensitive sarcoma cell lines (Fig. 3C and Table S4D). Given the repeated identification of the immune pathway, we utilized the CIBERSORTx program [27] to profile tumor-infiltrating immune cells in our sarcoma cell lines (Figure S4C and Table S4E). The results indicated significant variation in the proportions of immune cell types, particularly macrophages M2 and resting CD4 memory T cells. Most tissue samples exhibited a high proportion of macrophages M2, while the cell lines showed a high proportion of resting CD4 memory T cells. This finding suggests that the immune infiltration characteristics of the original sarcoma tissue were not well maintained in the corresponding cell lines, leading to the exclusion of immune profiles from further analysis.

**Fig. 4:**
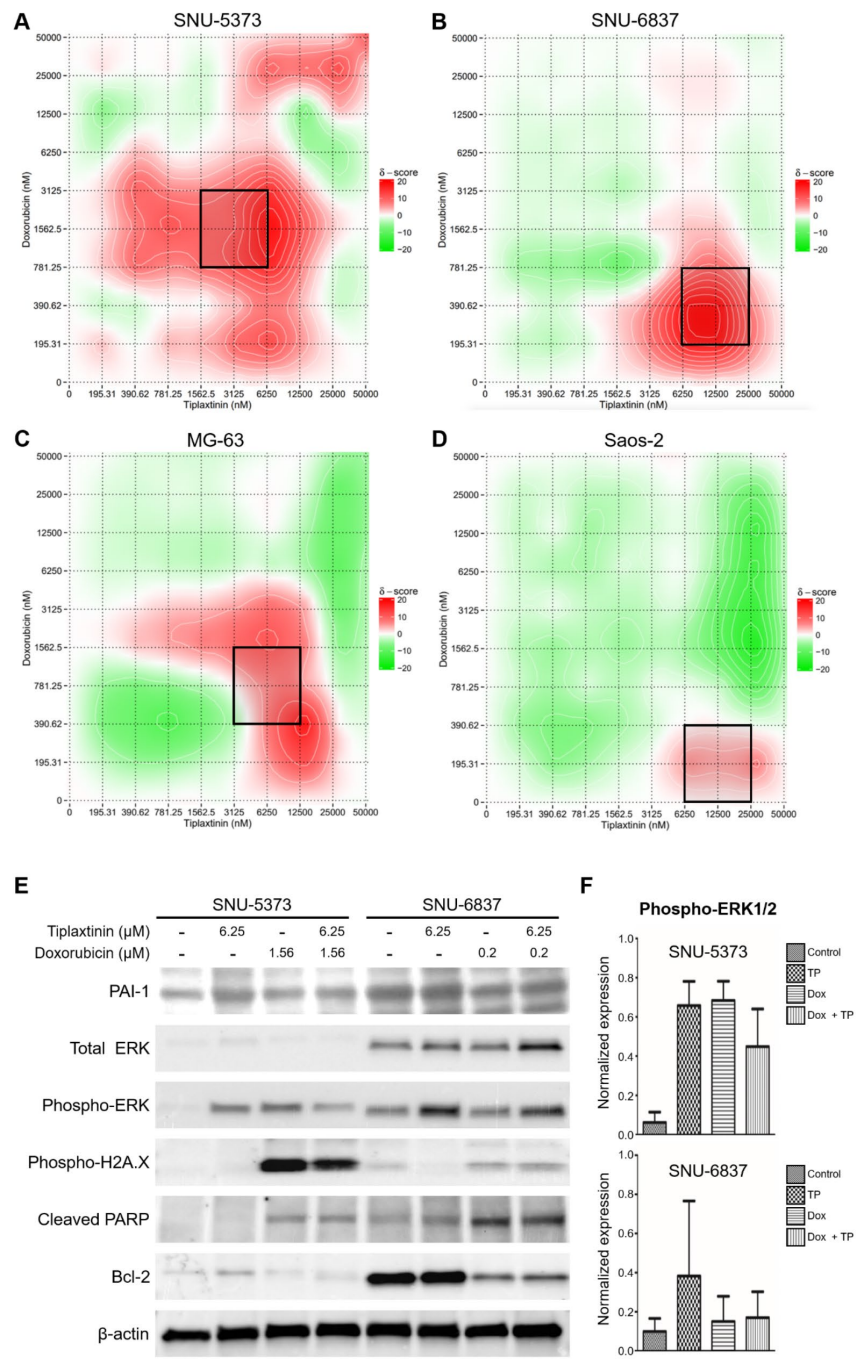
**Synergistic Effects of PAI-1 Inhibition and Doxorubicin in Modulating Doxorubicin Sensitivity in Sarcoma Cell Lines. See also Table S5A-D.** Heat maps illustrating the combination effects of Doxorubicin and Tiplaxtinin on four sarcoma cell lines: SNU-5373 (**A**), SNU-6837 (**B**), MG-63 (**C**), and Saos-2 (**D**). The x-axis represents the concentration of Tiplaxtinin (nM), and the y-axis shows the concentration of Doxorubicin (nM). The color scale indicates the synergy score, with red areas representing positive synergy (higher scores indicate stronger synergistic effects) and green areas indicating negative synergy. The black boxes highlight regions of significant synergy between the two drugs. **E.** Western blot analysis of ERK and phosphorylated ERK (pERK) protein levels in the SNU-6837 cell line. Samples were treated with 6.25 µM Tiplaxtinin, Doxorubicin, or a combination of both. β-actin is used as a loading control. The expected molecular weight for ERK is approximately 42–44 kDa, for pERK is approximately 42–44 kDa, and for β-actin is 42 kDa. **F.** Relative quantification of the Western blot bands from panel E, showing the levels of ERK and pERK in the SNU-6837 cell line under different treatment conditions. Quantification is normalized to the β-actin loading control. The y-axis represents normalized expression levels. Data are presented as mean ± standard error, demonstrating the differential activation of the ERK pathway in response to the combination therapy

Among several dysregulated pathways, the TGF-β signaling pathway was predominantly enriched in the doxorubicin-resistant group (Fig. 3D). Within this pathway, expressions of the SERPINE1 and MMP2 genes demonstrated a direct statistical correlation with the response to doxorubicin. Both genes were highly expressed in doxorubicin-resistant cell lines (Fig. 3E). Among these findings, survival analysis using the TCGA sarcoma database indicated that mRNA levels of SERPINE1 are significantly associated with patient survival (*p* < 0.01). Higher mRNA levels of SERPINE1 correlate with poorer patient prognosis, identifying SERPINE1 as a target gene related to Doxorubicin resistance (Figure S4D and S4E).

### Synergistic effects of PAI-1 and Doxorubicin in modulating Doxorubicin sensitivity

The synergistic effect of targeting the MMP2 gene in combination with Doxorubicin for treating osteosarcoma has been previously examined [28]. It was established that the MMP2 gene acts as an upstream regulator of Src kinase activity by inhibiting its endogenous suppressor, CHK/MATK, in osteosarcoma cells. However, the role of SERPINE1 in modulating Doxorubicin sensitivity in sarcoma treatment remains unexplored. To verify the expression levels of PAI-1 protein, we performed Western blot analysis using four public sarcoma cell lines and four SNU cell lines. Consistent with our earlier findings in transcriptomic analysis, Doxorubicin-resistant sarcoma cell lines overall exhibited elevated basal levels of PAI-1 in public and SNU cell lines except for Saos-2 cell line (Figure S5A). We further analyzed the protein expression of PAI-1 across different cellular compartments. Cytoplasmic PAI-1 displayed a consistent pattern, whereas nuclear PAI-1 levels varied in a cell line-specific manner. For instance, Doxorubicin-sensitive SNU-6179B and the Doxorubicin-resistant SNU-5373 cell lines exhibited higher PAI-1 expression. Doxorubicin-resistant cell lines showed elevated membranal PAI-1 expression. PAI-1 inactivates SMAD4 via the lipoprotein receptor-related protein 1 (LRP1) [29]. An inverse relationship was observed between nuclear PAI-1 and nuclear SMAD4 levels, aligning with previous findings. Cytoplasmic and membranal SMAD4 expressions were not closely correlated with SMAD4 levels (Figure S5B).

Subsequently, we utilized Tiplaxtinin, a PAI-1 inhibitor, to determine whether inhibiting PAI-1 enhances the cytotoxic effect of Doxorubicin in Doxorubicin-resistant cell lines. Tiplaxtinin demonstrated a high synergistic effect with Doxorubicin in these lines except for Saos-2 which expressed lower PAI-1 level. Notably, a midrange synergistic effect of both Doxorubicin and Tiplaxtinin was observed in the SNU-5373 cell line, indicating that both drugs contributed similarly to enhancing cytotoxicity (Fig. 4A and Table S5A). Conversely, a synergistic effect was noted at a lower dose of Doxorubicin and a higher dose of Tiplaxtinin in the SNU-6837 cell line, suggesting a greater cytotoxic impact when Doxorubicin was combined with Tiplaxtinin (Fig. 4B and Table S5B). MG-63 cell line exhibited similar pattern with SNU-6837. In contrast, Saos-2 showed lower synergistic score compared to other Sarcoma cell lines. This pattern was generally accordant with the level of PAI-1 validation the role of PAI-1 as an indicator of synergistic effect between Doxorubicin and Tiplaxtinin (Figure S5C).

Additionally, the SNU-6837 cell line exhibited a higher basal level of ERK protein. Treatment with 6.25 µM Tiplaxtinin increased the level of phosphorylated ERK (pERK), while Doxorubicin treatment had minimal effect on pERK levels (Fig. 4E and F). This indicates that Doxorubicin resistance in SNU-6837 may be associated with the MAPK/ERK pathway. The combination with Tiplaxtinin effectively countered the insensitivity by synergistically increasing pERK levels, explaining the higher dependency of the SNU-6837 cell line on Tiplaxtinin.

## Discussion

The establishment of new sarcoma cell lines is crucial in advancing our understanding of sarcoma biology, particularly in the context of drug resistance mechanisms. Currently, the field relies heavily on a limited number of widely used sarcoma cell lines, such as those available the Genomics of Drug Sensitivity in Cancer (GDSC) database [30]. While these cell lines have significantly contributed to sarcoma research, they present certain limitations that may hinder a comprehensive understanding of the disease’s heterogeneity. The public databases offer a valuable resource for high-throughput screening and genomic analyses; however, the sarcoma cell lines within these collections often lack the diversity needed to represent the full spectrum of sarcoma subtypes. These datasets include 66 widely used sarcoma cell lines that are subdivided into 15 sarcoma types. The most common type of sarcoma is Ewings sarcoma consisting 35% (*n* = 23), followed by osteosarcoma (15%, *n* = 10). In addition, many of these cell lines have been passaged extensively, leading to genetic drift and potentially reducing their relevance to the original tumor.

The newly established SNU sarcoma cell lines represent a broader range of sarcoma subtypes, including those that are underrepresented (Synovial sarcoma or Fibrosarcoma) or absent (Desmoplastic Small Round Cell Tumor) in existing collections. This diversity enhances the relevance of these cell lines in studying the heterogeneity of sarcomas and in developing subtype-specific therapies. Also, unlike many extensively passaged cell lines in the CCLE and GDSC, the SNU sarcoma cell lines have been carefully maintained to preserve their genomic integrity. Our study shows that these cell lines retain the genetic and transcriptomic characteristics of the primary tumors from which they were derived, providing a more accurate model for studying sarcoma biology and drug response. In addition, the SNU cell lines have been systematically characterized with a focus on doxorubicin resistance, one of the major challenges in sarcoma treatment. The comprehensive molecular profiling of these cell lines, including whole-exome and RNA sequencing, has provided insights into the genetic and transcriptomic factors that contribute to drug resistance. This makes them particularly valuable for investigating resistance mechanisms and testing new therapeutic strategies. In addition, few samples within our cohort represent spatial and temporal heterogeneity. Cell lines used in this study will be deposited in Korean Cell Line Bank (KCLB) and freely distributed worldwide through KCLB.

Our findings underscore the role of TP53 mutations and the differential expression of genes in pathways such as WNT/BETA-CATENIN and HEDGEHOG in modulating doxorubicin resistance. Furthermore, the integration of drug sensitivity data has enabled a more nuanced understanding of the relationship between cellular karyotype and chemotherapy efficacy, providing valuable insights that could drive the development of personalized therapeutic strategies for sarcoma patients.

Despite these advances, our study is not without limitations. The heterogeneity of sarcoma, both at the histological and genetic levels, presents significant challenges in fully capturing the diversity of resistance mechanisms. Moreover, the study’s reliance on in vitro cell line models may not entirely replicate the in vivo tumor microenvironment, potentially affecting the translatability of our findings to clinical settings. Additionally, while we have identified several key pathways associated with doxorubicin resistance, the functional roles of these pathways remain to be fully elucidated, necessitating further experimental validation.

Looking forward, the insights gained from this research lay a foundation for future studies aimed at overcoming doxorubicin resistance in sarcoma. One promising direction is the exploration of combination therapy strategies, such as the use of PAI-1 inhibitors like Tiplaxtinin, which have shown potential in enhancing doxorubicin efficacy through synergistic effects. Additionally, further research into the adaptive responses of sarcoma cells to prolonged doxorubicin exposure could reveal new mechanisms of acquired resistance, offering additional targets for therapeutic intervention. Ultimately, the goal is to translate these molecular insights into clinically actionable strategies that can improve treatment outcomes for sarcoma patients, emphasizing the need for continued research and collaboration in the field of cancer therapeutics.

## Electronic supplementary material

Below is the link to the electronic supplementary material.

Supplementary Material 1

Supplementary Material 2

## Data Availability

The datasets supporting the conclusions of this article will be available in the Sequence Read Archive (SRA) after acceptance. Few processed sequencing data will be available by request and governed by the Lead Contact. Computational pipelines as well as sequencing data including whole exome sequencing and RNA-sequencing in this study are available at the public repository: https://github.com/duddmswh12/Sarcoma/Scripts.

## References

[1] Damerell V, Pepper MS, Prince S. Molecular mechanisms underpinning sarcomas and implications for current and future therapy. Signal Transduct Target Ther. 2021;6:246. 34188019 10.1038/s41392-021-00647-8 PMC8241855

[2] Arifi S, Belbaraka R, Rahhali R, Ismaili N. Treatment of adult soft tissue sarcomas: an overview. Rare Cancers Ther. 2015;3:69–87. 27182479 10.1007/s40487-015-0011-x PMC4837937

[3] Penel N, Van Glabbeke M, Marreaud S, Ouali M, Blay JY, Hohenberger P. Testing new regimens in patients with advanced soft tissue sarcoma: analysis of publications from the last 10 years. Ann Oncol. 2011;22:1266–72. 21183581 10.1093/annonc/mdq608

[4] Grünewald TG, Alonso M, Avnet S, Banito A, Burdach S, Cidre-Aranaz F, Di Pompo G, Distel M, Dorado-Garcia H, Garcia-Castro J, et al. Sarcoma treatment in the era of molecular medicine. EMBO Mol Med. 2020;12:e11131. 33047515 10.15252/emmm.201911131 PMC7645378

[5] Das B, Jain N, Mallick B. piR-39980 mediates doxorubicin resistance in fibrosarcoma by regulating drug accumulation and DNA repair. Commun Biol. 2021;4:1312. 34799689 10.1038/s42003-021-02844-1 PMC8605029

[6] Mackall CL, Meltzer PS, Helman LJ. Focus on sarcomas. Cancer Cell. 2002;2:175–8. 12242149 10.1016/s1535-6108(02)00132-0

[7] Salawu A, Fernando M, Hughes D, Reed MW, Woll P, Greaves C, Day C, Alhajimohammed M, Sisley K. Establishment and molecular characterisation of seven novel soft-tissue sarcoma cell lines. Br J Cancer. 2016;115:1058–68. 27560552 10.1038/bjc.2016.259 PMC5117779

[8] Lee EY, Kim YH, Rayhan MA, Kang HG, Kim JH, Park JW, Park SY, Lee SH, You HJ. New established cell lines from undifferentiated pleomorphic sarcoma for in vivo study. BMB Rep. 2023;56:258–64. 36789562 10.5483/BMBRep.2022-0209 PMC10140480

[9] May WA, Grigoryan RS, Keshelava N, Cabral DJ, Christensen LL, Jenabi J, Ji L, Triche TJ, Lawlor ER, Reynolds CP. Characterization and drug resistance patterns of Ewing’s sarcoma family tumor cell lines. PLoS ONE. 2013;8:e80060. 24312454 10.1371/journal.pone.0080060 PMC3846563

[10] Fujii H, Honoki K, Tsujiuchi T, Kido A, Yoshitani K, Takakura Y. Sphere-forming stem-like cell populations with drug resistance in human sarcoma cell lines. Int J Oncol. 2009;34:1381–6. 19360350

[11] Eulo V, Van Tine BA. Immune checkpoint inhibitor resistance in soft tissue sarcoma. Cancer Drug Resist. 2022;5:328–38. 35800372 10.20517/cdr.2021.127 PMC9255245

[12] Porcelli L, Garofoli M, Di Fonte R, Fucci L, Volpicella M, Strippoli S, Guida M, Azzariti A. The β-adrenergic receptor antagonist propranolol offsets resistance mechanisms to chemotherapeutics in diverse sarcoma subtypes: a pilot study. Sci Rep. 2020;10:10465. 32591592 10.1038/s41598-020-67342-6 PMC7320177

[13] Mills J, Matos T, Charytonowicz E, Hricik T, Castillo-Martin M, Remotti F, Lee FY, Matushansky I. Characterization and comparison of the properties of sarcoma cell lines in vitro and in vivo. Hum Cell. 2009;22:85–93. 19874397 10.1111/j.1749-0774.2009.00073.x PMC3000410

[14] Barretina J, Taylor BS, Banerji S, Ramos AH, Lagos-Quintana M, Decarolis PL, Shah K, Socci ND, Weir BA, Ho A, et al. Subtype-specific genomic alterations define new targets for soft-tissue sarcoma therapy. Nat Genet. 2010;42:715–21. 20601955 10.1038/ng.619 PMC2911503

[15] Forbes SA, Bindal N, Bamford S, Cole C, Kok CY, Beare D, Jia M, Shepherd R, Leung K, Menzies A, et al. COSMIC: mining complete cancer genomes in the catalogue of somatic mutations in Cancer. Nucleic Acids Res. 2011;39:D945–950. 20952405 10.1093/nar/gkq929 PMC3013785

[16] Soong D, Stratford J, Avet-Loiseau H, Bahlis N, Davies F, Dispenzieri A, Sasser AK, Schecter JM, Qi M, Brown C, et al. CNV Radar: an improved method for somatic copy number alteration characterization in oncology. BMC Bioinformatics. 2020;21:98. 32143562 10.1186/s12859-020-3397-x PMC7060549

[17] Pertea M, Pertea GM, Antonescu CM, Chang T-C, Mendell JT, Salzberg SL. StringTie enables improved reconstruction of a transcriptome from RNA-seq reads. Nat Biotechnol. 2015;33:290–5. 25690850 10.1038/nbt.3122 PMC4643835

[18] Ianevski A, Giri AK, Aittokallio T. SynergyFinder 3.0: an interactive analysis and consensus interpretation of multi-drug synergies across multiple samples. Nucleic Acids Res. 2022;50:W739–43. 35580060 10.1093/nar/gkac382 PMC9252834

[19] Seddon B, Strauss SJ, Whelan J, Leahy M, Woll PJ, Cowie F, Rothermundt C, Wood Z, Benson C, Ali N, et al. Gemcitabine and Docetaxel versus doxorubicin as first-line treatment in previously untreated advanced unresectable or metastatic soft-tissue sarcomas (GeDDiS): a randomised controlled phase 3 trial. Lancet Oncol. 2017;18:1397–410. 28882536 10.1016/S1470-2045(17)30622-8 PMC5622179

[20] Tanaka K, Machida R, Kawai A, Nakayama R, Tsukushi S, Asanuma K, Matsumoto Y, Hiraga H, Hiraoka K, Watanuki M, et al. Perioperative Adriamycin plus Ifosfamide vs. gemcitabine plus docetaxel for high-risk soft tissue sarcomas: randomised, phase II/III study JCOG1306. Br J Cancer. 2022;127:1487–96. 35871234 10.1038/s41416-022-01912-5 PMC9553903

[21] McGovern Y, Zhou CD, Jones RL. Systemic therapy in metastatic or Unresectable Well-Differentiated/Dedifferentiated Liposarcoma. Front Oncol. 2017;7:292. 29250486 10.3389/fonc.2017.00292 PMC5715199

[22] Kim JH, Park HS, Heo SJ, Kim SK, Han JW, Shin KH, Kim SH, Hur H, Kim KS, Choi YD, et al. Differences in the efficacies of Pazopanib and Gemcitabine/Docetaxel as Second-Line treatments for metastatic soft tissue sarcoma. Oncology. 2019;96:59–69. 30336470 10.1159/000492597

[23] Yang W, Soares J, Greninger P, Edelman EJ, Lightfoot H, Forbes S, Bindal N, Beare D, Smith JA, Thompson IR, et al. Genomics of Drug Sensitivity in Cancer (GDSC): a resource for therapeutic biomarker discovery in cancer cells. Nucleic Acids Res. 2013;41:D955–961. 23180760 10.1093/nar/gks1111 PMC3531057

[24] Warren A, Chen Y, Jones A, Shibue T, Hahn WC, Boehm JS, Vazquez F, Tsherniak A, McFarland JM. Global computational alignment of tumor and cell line transcriptional profiles. Nat Commun. 2021;12:22. 33397959 10.1038/s41467-020-20294-x PMC7782593

[25] Watson EV, Lee JJ, Gulhan DC, Melloni GEM, Venev SV, Magesh RY, Frederick A, Chiba K, Wooten EC, Naxerova K, et al. Chromosome evolution screens recapitulate tissue-specific tumor aneuploidy patterns. Nat Genet. 2024;56:900–12. 38388848 10.1038/s41588-024-01665-2 PMC11096114

[26] Liberzon A, Birger C, Thorvaldsdóttir H, Ghandi M, Mesirov JP, Tamayo P. The Molecular signatures database (MSigDB) hallmark gene set collection. Cell Syst. 2015;1:417–25. 26771021 10.1016/j.cels.2015.12.004 PMC4707969

[27] Steen CB, Liu CL, Alizadeh AA, Newman AM. Profiling cell type abundance and expression in bulk tissues with CIBERSORTx. Methods Mol Biol. 2020;2117:135–57. 31960376 10.1007/978-1-0716-0301-7_7 PMC7695353

[28] Maybee DV, Cromwell CR, Hubbard BP, Ali MAM. MMP-2 regulates Src activation via repression of the CHK/MATK tumor suppressor in osteosarcoma. Cancer Rep (Hoboken). 2023;7:e1946. 38064181 10.1002/cnr2.1946 PMC10849928

[29] Lin LL, Kost ER, Lin CL, Valente P, Wang CM, Kolonin MG, Daquinag AC, Tan X, Lucio N, Hung CN, et al. PAI-1-Dependent inactivation of SMAD4-Modulated Junction and Adhesion Complex in obese endometrial Cancer. Cell Rep. 2020;33:108253. 33053339 10.1016/j.celrep.2020.108253 PMC7641039

[30] De Vita A, Mercatali L, Miserocchi G, Liverani C, Spadazzi C, Recine F, Bongiovanni A, Pieri F, Cavaliere D, Fausti V et al. Establishment of a primary culture of patient-derived soft tissue Sarcoma. J Vis Exp 2018. 10.3791/56767 PMC5933451 29708525

