# Supplementary material for: Establishment and characterization of 18 Sarcoma Cell Lines: Unraveling the Molecular Mechanisms of Doxorubicin Resistance in Sarcoma Cell Lines
Source: J Transl Med. 2024 Oct 2;22:889. doi: 10.1186/s12967-024-05700-y (PMC11445991; doi:10.1186/s12967-024-05700-y)
Supplement: Supplementary file 1 — Supplementary Material 1 [file 12967_2024_5700_MOESM1_ESM.docx]

**Supplementary Figures**


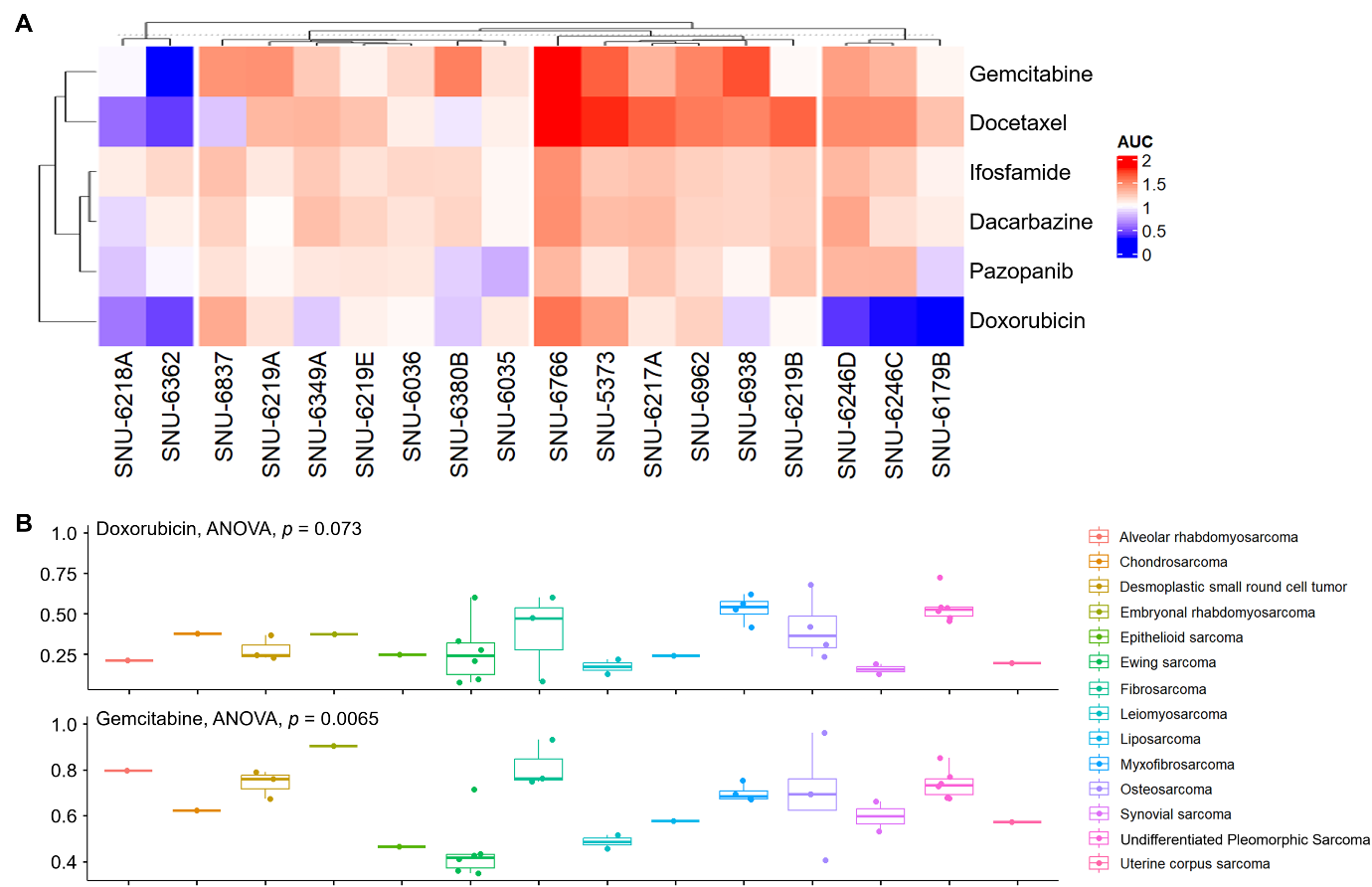


**Figure S1. Drug Response Profiles of Sarcoma Cell Lines A.** Screening of 18 established SNU sarcoma cell lines with six clinically used drugs (Dacarbazine, Docetaxel, Doxorubicin, Gemcitabine, Ifosfamide, and Pazopanib). Drug response profiles are illustrated, with color coding to indicate the level of sensitivity (Red: poorer response, Blue: better response). **B.** Boxplot comparison of drug response data for Doxorubicin and Gemcitabine across various sarcoma subtypes, as obtained from the Genomics of Drug Sensitivity in Cancer (GDSC) database. The boxplots display the range of sensitivity (Area Under the Curve, AUC) to Doxorubicin (top) and Gemcitabine (bottom) among different sarcoma subtypes. Each sarcoma subtype is represented by a distinct color, as indicated in the legend. The p-values from ANOVA tests are provided to assess the statistical significance of the differences in drug sensitivity among the subtypes.


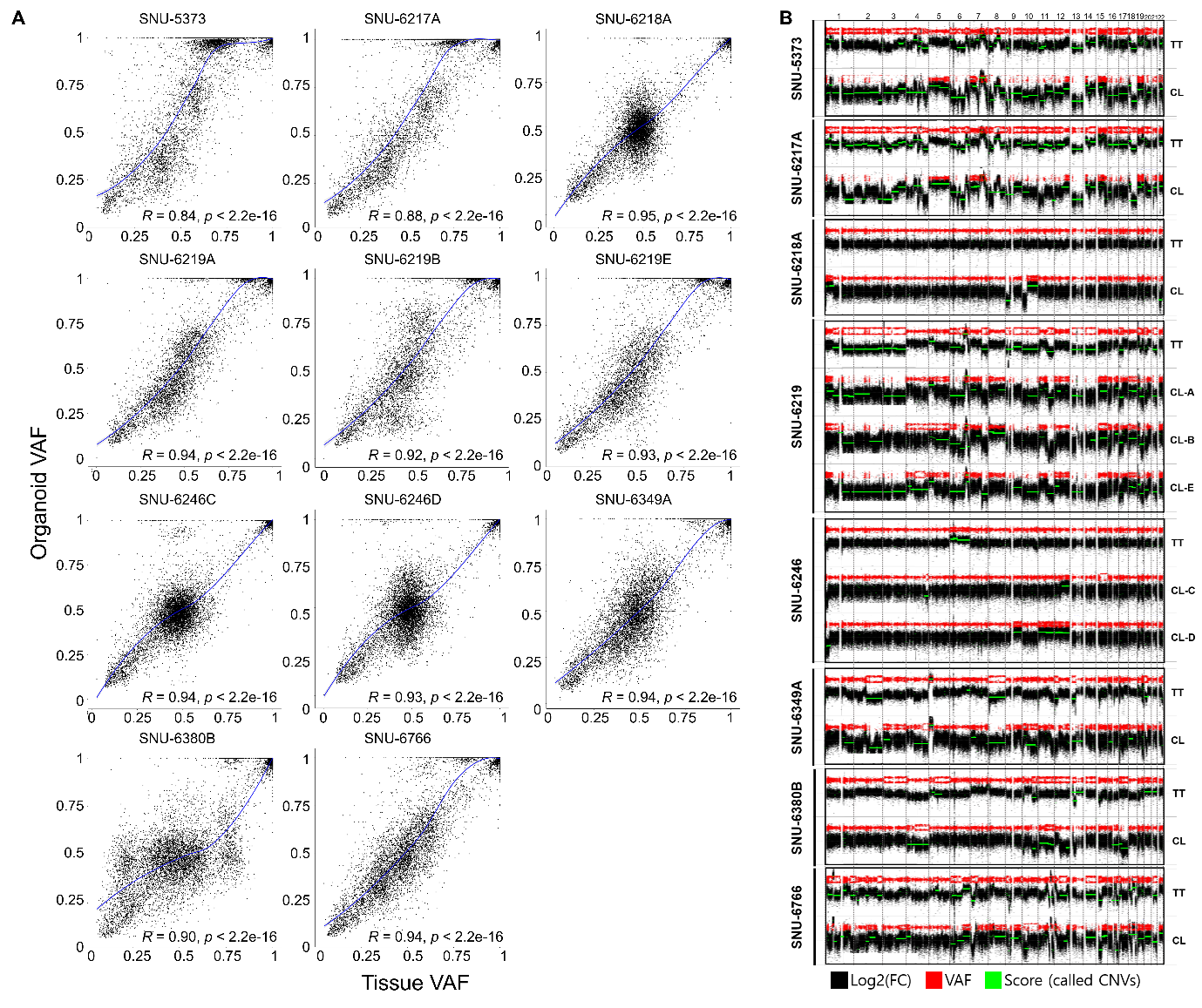


**Figure S2. Sensitivity and Genetic Correlation of Sarcoma Cell Lines to Doxorubicin. A.** Comparative analysis of variant allele frequencies (VAFs) between tumor tissues and cell lines, showing high correlation coefficients (R > 0.84). X-axis indicates Tissue VAFs and Y-axis represents Cell Lines VAFs. The pattern of correlation is indicated with blue line. **B.** Detailed CNV analysis of cell lines and original tumor tissues. The figure displays log2 fold change (black), variant allele frequency (red), and copy number variations (green).


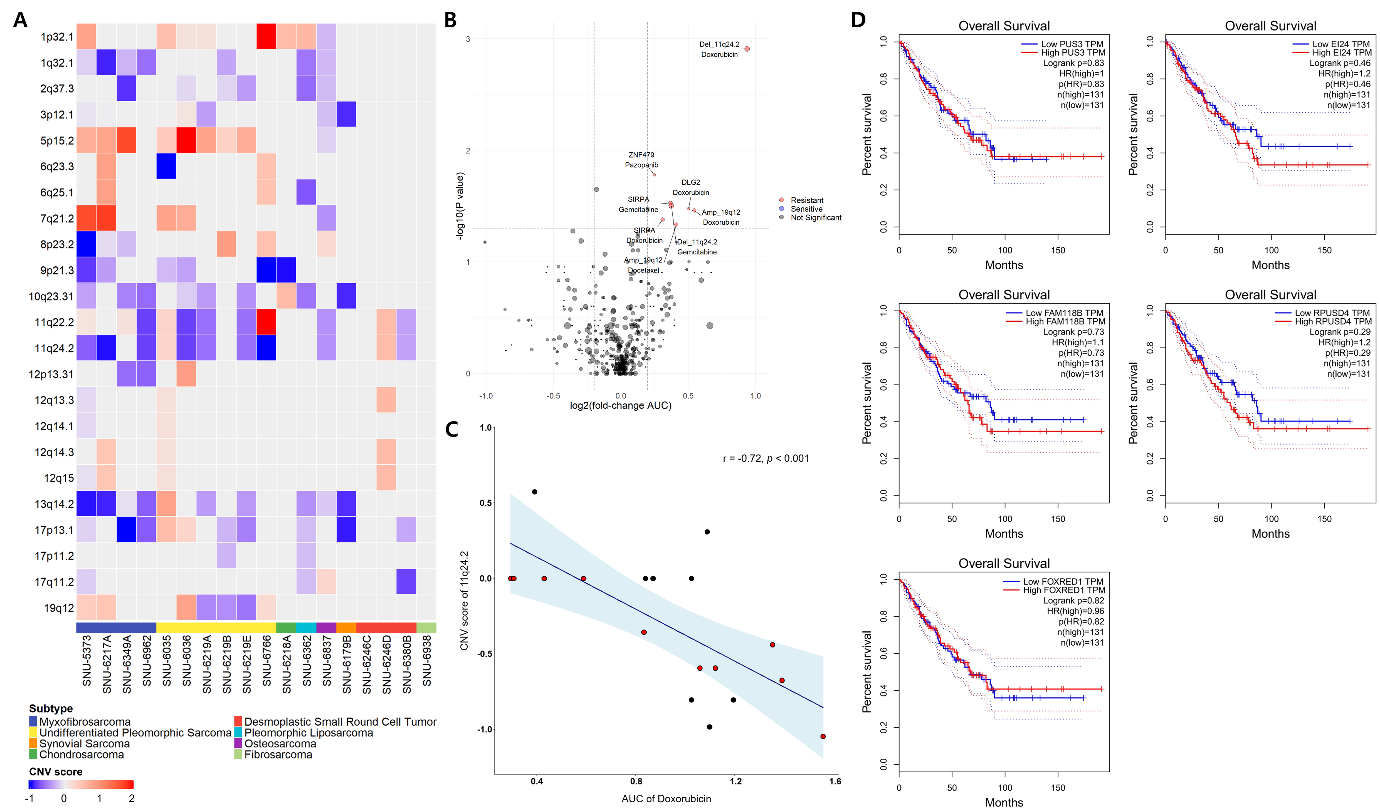


**Figure S3. Correlation Between CNV at 11q24.2 and Doxorubicin Resistance. A.** CNV analysis of frequently aberrated genomic loci in the SNU sarcoma cell line cohort. The panel presents CNV patterns across various sarcoma subtypes, with each subtype marked by representative colors. Specific loci, including 11q24.2, are highlighted. **B.** Gene-drug interaction analysis displaying the relationship between CNV deletions and drug response. The panel illustrates significant interactions identified using the Wilcoxon rank-sum test. CNV deletions, including those at 11q24.2, are highlighted. **C.** Pearson correlation analysis between the CNV score at the 11q24.2 locus and the AUC values for Doxorubicin response. The panel shows the distribution of data points, with correlations within the 95% confidence interval marked with red dots. **D.** Survival analysis using the TCGA sarcoma database, depicting the association between mRNA expression of genes located at the 11q24.2 locus (including PUS3, EI24, FAM118B, RPUSD4, and FOXRED1) and patient prognosis. Kaplan–Meier analysis was performed using a threshold of p > 0.2.


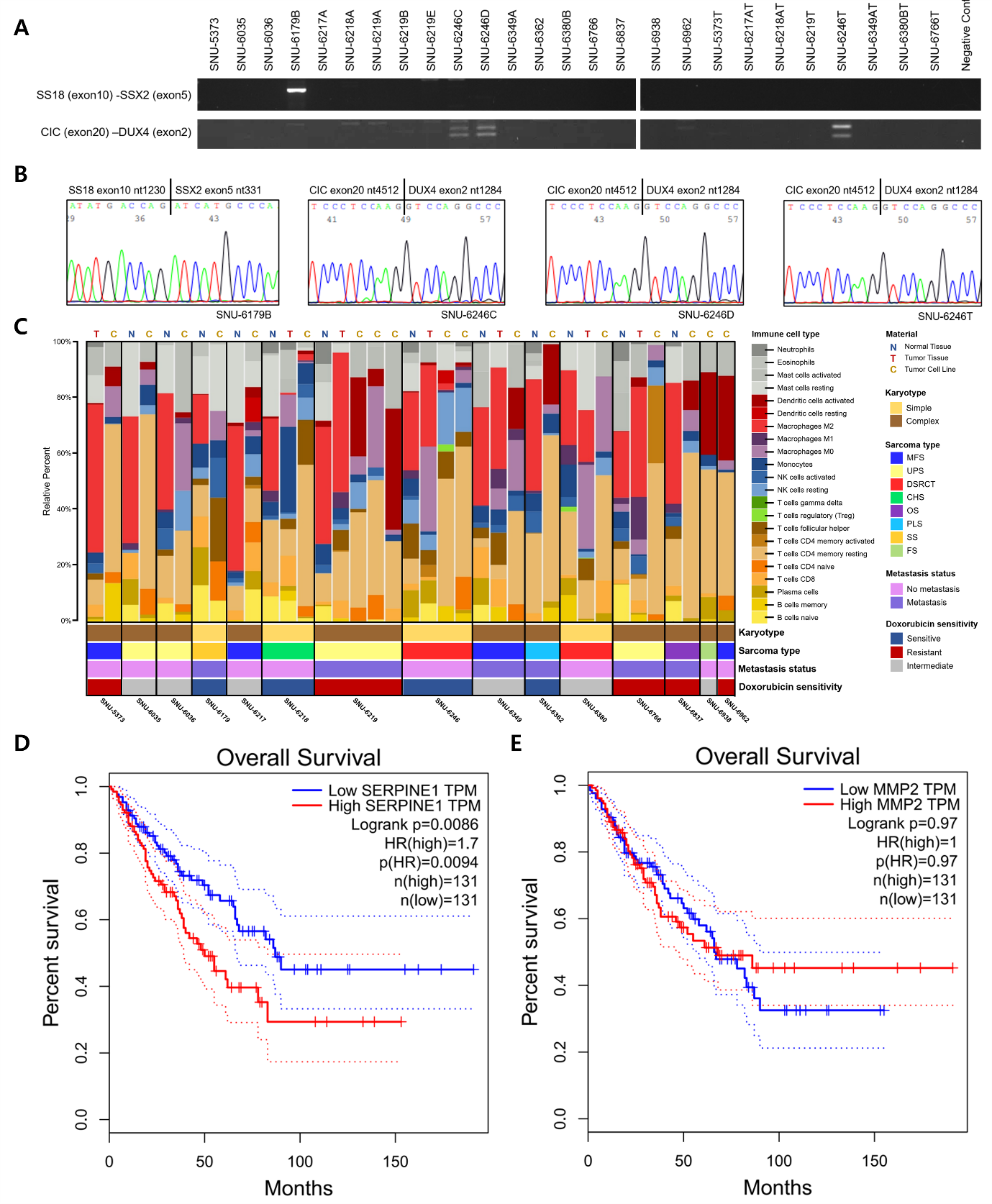


**Figure S4. Fusion Gene Identification and Immune Cell Profiling in Sarcoma Cell Lines. A.** RNA sequencing identifies the SS18-SSX2 fusion gene in SNU-6179B and CIC-DUX4 fusion gene in SNU-6246C and SNU-6246D. B. Sanger sequencing confirmed the fusion break junction of SS18-SSX2 fusion gene in SNU-6179B and CIC-DUX4 fusion gene in SNU-6246C, SNU-6246D and SNU-6246T. **C.** CIBERSORTx analysis profiles tumor-infiltrating immune cells in sarcoma cell lines, showing significant variation in immune cell proportions between tissue samples and cell lines. Most tissue samples exhibit high proportions of macrophages M2, while cell lines show high proportions of resting CD4 memory T cells. Each immune cell type is indicated with representative colors. Classifying factors including karyotype, sarcoma type, metastasis status, and doxorubicin sensitivity are indicated below. **D.** Survival analysis using the TCGA sarcoma database indicates that higher mRNA levels of SERPINE1 are significantly associated with poorer patient prognosis (p < 0.01). **E.** Survival analysis using the TCGA sarcoma database indicates that mRNA expression of MMP2 are rarely associated with patient prognosis (p < 0.97).


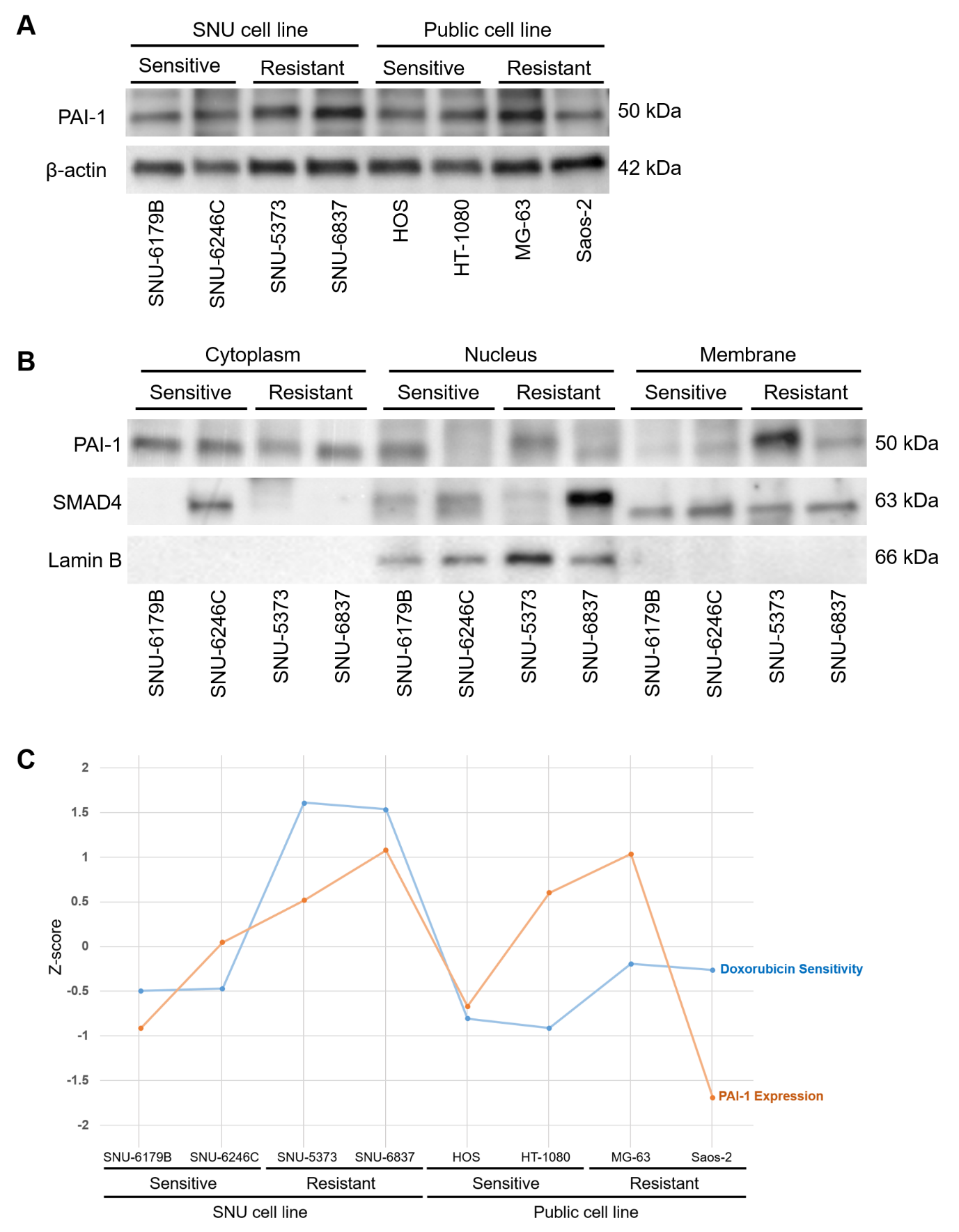


**Figure S5. Analysis of PAI-1 expression and subcellular localization in sarcoma cell lines with varying Doxorubicin sensitivity.** **A.** Western blot analysis of PAI-1 protein expression in SNU cell lines and public cell lines categorized by Doxorubicin sensitivity (sensitive vs. resistant). β-actin serves as a loading control. **B.** Analysis of SERPINE1 (PAI-1) protein expression across different cellular compartments in sarcoma cell lines. Cytoplasmic, nuclear, and membranal PAI-1 levels are shown. **C.** Line graph displaying the relationship between Doxorubicin sensitivity (blue line) and PAI-1 expression (orange line) across various cell lines. The x-axis lists the cell lines divided into sensitive and resistant categories, while the y-axis shows the Z-score for both Doxorubicin sensitivity and PAI-1 expression.
